# Obstacles to continuity of care in young mental health service users' pathways - an explorative study

**Published:** 2013-08-29

**Authors:** Marian Ådnanes, Sissel Steihaug

**Affiliations:** SINTEF Technology and Society, Department of Health Research, Trondheim, Norway; SINTEF Technology and Society, Department of Health Research, Oslo, Norway; HØKH, Research Centre, Akershus University Hospital, Lørenskog, Norway

**Keywords:** mental health services, continuity of care, care pathways, obstacles, young adults

## Abstract

**Background:**

Users of mental health services often move between different primary and specialised health and care services, depending on their current condition, and this often leads to fragmentation of care. The aim of this study was to map care pathways in the case of young adult mental health service users and to identify key obstacles to continuity of care.

**Method:**

Quarterly semi-structured interviews were performed with nine young adults with mental health difficulties, following their pathways in and out of different services in the course of a year.

**Results:**

Key obstacles to continuity of care included the mental health system's lack of access to treatment, lack of integration between different specialist services, lack of progress in care and inadequate coordination tools such as ‘Individual Plan’ and case conferences that did not prevent fragmented care pathways.

**Conclusions:**

Continuity of care should be more explicitly linked to aspirations for development and progress in the users' care pathways, and how service providers can cooperate with users to actually develop and make progress. Coordination tools such as case conferences and ‘individual plans’ should be upgraded to this end and utilised to the utmost. This may be the most effective way to counteract the system obstacles.

## Introduction

Mental health care pathways have changed radically as a consequence of deinstitutionalisation. Tasks previously performed by centralised psychiatric institutions are now handled by a range of primary and secondary health and care providers. Users of mental health services often move between different services, depending on their current condition, and this often leads to fragmentation of care. In this study, we will explore the movement and progress of young adult users within mental health services in order to identify obstacles to continuity of care.

Continuity of care has become an important aspect of the provision of mental health services, and primarily involves coordination of the user's progress through the system [1–3]. The definitions of continuity of care are multivariate, emphasizing many circumstances that affect progress in a pathway. Bachrach provided a thorough review of the concept 30 years ago, defining continuity of care as the orderly, uninterrupted movement of patients among the diverse elements of the health delivery system [1]. Later definitions include social and psychological elements; the degree to which a series of discrete health care events is experienced as coherent, connected and consistent with the user's health care needs and personal context [4] and the experience of progression of care from the patient's point of view [5]. However, efforts to describe problems or formulate solutions related to enhancing continuity of care are considered to be complicated because of the lack of a clear definition [6,7] or of consensus on the definition [3,4]. A confounding influence of individual user characteristics on the relationship between service process and outcome contributes to the confusion [3].

Several studies have operationalised continuity of care, including the users' experience of the services provided [5–14] and identified a number of prerequisites for achieving continuity of care; access to services, stable services without breaks, the same member of staff being seen, coordination between different health providers, etc. The core of this research has been the study of the nature and operation of the concept of continuity in order to explore the extent to which the services provided are actually characterised by continuity and their usefulness to their users. Studies show that continuity of care has psychological and social effects in terms of the user's quality of life, ability to function within the community and satisfaction with the care services [14], and also effects on the system, for example, through lower hospital costs and higher community costs [15]. Continuity of care may be considered as both a process and an outcome [16], but in terms of individual patient outcome, the results tend to be more variable [14,17,18.

The Norwegian health care system is divided into two separate governmental levels, with hospitals organised within the specialist health care system, while the municipalities hold the responsibility for providing primary health care and social services, including primary mental health care services. The specialised mental health services are to be integrated with and run according to the same principles as other specialised health care services. The two levels act in accordance with different laws and regulations, and the coordination between them is a challenge. In the mental health field, challenges resulting from fragmentation of care have been focused and worked with in parallel with the deinstitutionalisation. A green paper acknowledged failures at all levels [19] and was followed by a 10-year national Mental Health Escalation Program (1998–2008) targeting the services [20]. The evaluation of the program showed that while many of the quantitative goals for the mental health services had been reached, much remained to be improved in terms of the quality of services, especially in terms of coordination and collaboration between them [21].

To facilitate comprehensive and coordinated care pathways, the main goal in the ongoing Coordination Reform in Norway is to ensure that the user of health services receives the right treatment at the right time and on the right service level [22], a key international health policy goal as well. In the Norwegian reform document a pathway is defined as ‘the chronological chain of events that make up patients meeting with different health-care systems’ [22, p. 15]. Good pathways are characterised in that ‘these events are put together in an efficient and coordinated manner to meet the patient's individual needs’ [22, p. 15].

The aim of this study is to map care pathways in the case of young adult mental health service users and to identify key obstacles to continuity of care.

## Methods

Quarterly semi-structured interviews, 32 in total, were carried out with nine young adults through a one-year data-collection period. The informants were between 18 and 30 years of age with one or several mental health diagnoses, some also with substance abuse, all of them with relatively complex needs for treatment and follow-up.

## Sample

Invitations to participate were directed to users via clinicians at three district psychiatric centres, one hospital department and mental health services in two municipalities. Additional invitations were issued via a user organisation (information presented at members' meeting and a sign on the information board) and a secondary school for young adults with mental health problems (sign on the information board). Three informants were recruited via municipal services, one via the user organisation, three via the secondary school, and two were recruited via informants already recruited. Finally, eight women and one man signed the informed consent form, indicating they were willing to be interviewed four times during one year.

Five of our nine informants told they were diagnosed with severe mental illness: four with personality disorder and one with manic depression. Three of those without a severe mental illness had been diagnosed with depression, and one had not yet been diagnosed. Four of the nine had post-traumatic stress disorder, and three had an eating disorder. All of them mentioned anxiety as a more or less severe problem in periods. Three presently had major problems with alcohol, tablet and/or substance abuse; three had repeated problems with acts of self-harm and eight had taken an overdose once or several times or attempted other suicidal acts. While two of the informants were relatively new receivers of mental health services, seven had long careers ranging from 8 to 16 years of contact.

Eight of the informants received work assessment allowance, one in combination with disability insurance and one in combination with a part-time job. Seven of them took courses at secondary school, trying to qualify for higher studies and one studied at college. One had finished her education as an enrolled nurse, but started a new education at the end of the data collection.

The first author carried out the interviews, audiotaped and transcribed them. The interviews focused on: the persons' history of mental health and service-use since their first encounter with mental health services, self-perceived mental and somatic health situation, situation in terms of housing, work, education, and daily activities, contact with health and welfare services; type and amount of contact, need and accessibility, and experience of the services.

The interviews lasted from 30 to 90 minutes (mean 50 minutes). Four interviews were carried out with each informant, except for two who dropped out before all interviews were performed. The transcripts of interviews consisted of 192 pages (single-spaced).

## Analysis

**Figure 1.**
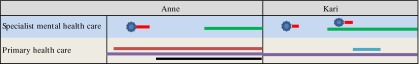
Pathways of type-1 informants (relatively recent mental health problems and needs for treatment).

**Figure 2.**
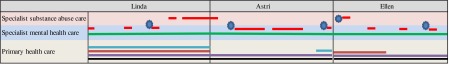
Pathways of Type-2 informants (long-lasting mental health and substance abuse problems and constant change of treatment services).

**Figure 3.**
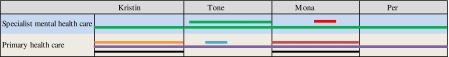
Pathways of Type-3 informants (long-lasting mental health problems, but without substance abuse problems and with stable treatment).

The interview transcripts constitute the data in this study. First, the informants' information given in the interviews about the services involved and at what time they were involved during the study period (one year), formed the basis for a schematic presentation of each informant's care pathway (Figures 1–3).

Furthermore, in order to identify obstacles to continuity in each informant's pathway, the interview transcripts were analysed qualitatively. The procedure for analysis is inspired by principles of grounded theory and in line with Crabtree and Miller's strategy of editing analysis style [23]. This strategy is partly guided by data and partly by previous research and theory, here on the topic of continuity of care. The transcriptions were first read through several times by both authors in order to create an overall impression. Second, each interview was analysed lengthwise, identifying meaningful units and segments in the text describing important events that affected their care pathway in a way that led to violations. These events were coded and categorised, and organised into explanatory themes under the following headlines: treatment, illness, medication, substance abuse, local services and coordinating tools. From these themes, quotations describing the various forms of violations in each informant's care pathway were selected. In the final interview with each informant the interviewer went through the mapping of services, the historic time-line and important events in the informant's care pathway in order to validate the data.

## Ethics

Ethical approval was obtained in advance from the Regional Committee for Medical and Health Research Ethics (ref.: 2010/1144). Informed consent was obtained from the participants before the interviews. Principles of confidentiality and anonymity have been applied in the conduct, reporting and storage of data arising from this study in accordance with the act on processing of personal information and the requirements of the Regional Ethics committee, based on the Health Research Act.

## Methodological considerations

The sample was somewhat biased. First, eight of the informants were either full- or part-time students, four of them at a secondary school for young people with mental health problems. This, together with their relatively clear situations in terms of social security, housing, etc., identifies them as a more resourceful group of users than the average mental health service user within the same age group and with the same diagnoses and difficulties. Furthermore, it is likely that the group of informants was biased simply based on their willingness to participate. For example, it is probable that they were more interested in influencing decisions related to their own treatment and care and services. Finally, the sample was made up of eight women and one man. In an exploratory study such as this, whose aim was to identify obstacles within the system (but not their prevalence), we simply have to take into account that there is a lack of data about men.

Most studies of continuity of care include persons with long-term psychotic disorders because fragmented pathways are assumed to be more of a problem among such service users. However, our study included participants with both severe and less-severe mental illnesses. Catty et al. [24] show that ‘non-psychotic service users’ are at even greater risk of experiencing disruptive and distressing care transitions and should, therefore, be included in studies of continuity of care. In terms of diagnoses, however, compared to the total number of informants we have many with personality disorder and many with post-traumatic stress disorder.

The particular strength of our study is that informants were followed throughout an entire year, with quarterly interviews, a design that provided reliable information about the informants' pathway, as a retrospective design that relied on the informant's memory would not have done. Furthermore, it allowed data to be checked and any misunderstandings to be resolved. The data and findings were also discussed with a reference group of senior researchers from several research institutes and universities.

## Results

The informants' care pathways can be broadly classified into three typologies based on the following characteristics: duration of mental health difficulties, presence of simultaneous mental disorders and substance abuse problems and the stability of services received. Type 1: relatively recent mental health problems and needs for treatment; Type 2: long-lasting mental health and substance abuse problems and constant change of treatment services; Type 3: long-lasting mental health problems, but without substance abuse problems and with stable treatment.

Figures 1–3 below describe the informants' care pathways in a schematic timeline through one year. It specifies when and for how long the various services were involved as well as emergency hospitalisations associated with overdoses/suicide attempts. Table 1 provides an overview of the colour categories for the various services in Figures 1–3.

Among the informants whose need for treatment for mental health problems was of recent origin, the central issue was that of obtaining support and treatment rapidly. There tended to be two main causes of breaks in their pathways; a long interval between the need for support emerging and the availability of treatment, and a long wait for a decision regarding further treatment after their first stay in hospital. In both cases, the waiting time for treatment was shortened in the light of actions that suggested that both were in danger of attempting suicide. The need for treatment was both obvious and urgent. Kari was less than 18 years old when she took her first overdose, and she was taken under the care of the child and adolescent psychiatric service. Anne had a well-established offer from the local mental health care service, with weekly follow-ups and a regular contact person even before her application for treatment. The follow-up procedure consisted largely of organising practical aspects of her problem: applying for a treatment place in the specialist mental health service, submitting reminders about the application and introducing her to low-threshold day care. Anne's long-lasting somatic health problem, and her consequent need for constant care services probably helped to ensure that an ‘Individual Plan’ was rapidly implemented (‘Individual Plan’ is a statutory right for persons in need of long-term, coordinated health services and intended as a tool to ensure that services and measures work and are experienced as unified, coordinated and individually adapted to each patient). In Kari's case, the ambulant substance abuse and mental health team became involved following her second overdose.

Figure 2 illustrates the pathways of the Type-2 informants, who were characterised by their multitude of problems: mental health and addiction problems, eating disturbances, self-harm and overdosing. Their treatment pathways are characterised by frequent transfers between services partly because their individual problems have been treated separately by different specialist health services. This applies to measures aimed directly not only at addiction and mental health but also to the fact that specialised treatment measures may be distinct from those directed towards eating disturbances and exclude other mental health problems. The fact that the treatments provided helped to improve the users' situation to different degrees was a contributory cause of the changes. Some of the emergency hospital admissions were in response to overdoses and, in some cases, were followed by prolonged hospital stays. All three users were also continuously followed-up by the specialist mental health care services. Linda was also closely followed up by the local mental health services, at primary care level, both via a regular contact person and by relatively regular contact with the ambulant substance abuse and mental health team. Ellen as well had a regular contact person in the primary care mental health unit, but had more or less dropped this contact. Astri only had contact with her general practitioner. In all three cases, the informants' services met in so-called case conferences (meetings involving representatives of the services and the user in order to coordinate efforts and clarify responsibilities), but the meetings were too seldom with the constant new requirements and situations that emerged in the pathways.

Figure 3 illustrates the pathways of the Type-3 informants, who were characterised by their long periods in stable treatment conditions, which involved the same treatment provider or contact person. One core difference from the Type-2 informants was that this group had no addiction issues. Three of them had a continuous and long-duration pathway, while two (Kristin and Per) experienced a gradual reduction in their contact with their treatment provider. While Tone and Per only had contact with the primary care mental health service, they did not believe that that they needed any coordinating measure like ‘Individual Plan’ or a case conference. In Tone's case, this was because her two treatment providers communicated well with each other. In Per's, because no services other than that of the treatment provider at the district psychiatric centre were involved. In Per's case, attempts were made to involve his general practitioner to a greater extent, but without success. Mona differed from the others in that she had not had a regular treatment programme with the specialist service during the past few years, but was regularly followed up by the primary care mental health service, and was treated as a psychiatric in-patient in periods when her symptoms became more severe. After a long time, she asked, during a case conference, for a regular treatment programme that could help her to make progress. On her request, she was then referred to outpatient treatment within the specialist service and a treatment programme that comprised cognitive therapy, coping techniques and reduction in medication intake.

## Discussion

We have explored care pathways throughout a period of one year in a group of young adult users of mental health services. Key obstacles to continuity of care were caused by the system's lack of access to treatment, lack of integration between different specialist services, lack of progress in care and inadequate coordination tools unable to prevent fragmented care pathways.

## Lack of access to treatment

Among the informants whose need for treatment had emerged relatively recently, the long waiting time for specialist treatment hindered not only their progress but also resulted in deterioration of their condition, thoughts of suicide and overdosing.

Accessibility and continuity are closely related [1,7]. Without easy access to a range of services, the user cannot be helped by the system to progress and continuity of care cannot be in place [8]. The importance of access to treatment as a separate dimension of the continuity concept naturally varies with the length of wait for treatment. It is tempting to assume, particularly when the waiting time for specialist services is long, that treatment measures within primary care services could have offered a more appropriate and effective pathway, but this is dependent on the services offered in the community setting. In Norway, the range of services varies widely between local authorities [25,26], as do attitudes among professionals and users, to primary care services as an arena for treatment and what treatment involves. In this field, there is a steadily growing tension between traditional scientific and contextual approaches in Norway [27], something that is reflected in the processes involved in the design of municipal mental health services [28]. This is also reflected in different treatment philosophies and competence between local bed units and central bed units within specialised services [29]. Reforms and measures for more decentralised services in Norway and in other countries aim at changing the division of labour between specialist care and primary care through the expansion of responsibility and involvement, for example, via ambulant mental health teams (home treatment, crisis resolution, etc.) and various forms of low-threshold treatment. Thornicroft and Tansella [30] argue in favour of ‘balanced care’, focusing on services provided in normal community settings with admissions to hospital only when necessary. Their review of the question of how far mental health services should be provided in community and/or hospital settings supports this balanced approach in that they found no evidence in favour of relying on hospital services alone, nor that community services by themselves can provide satisfactory and comprehensive care [31].

The fact that the informants in our study were primarily concerned about access to treatment and not concerned about social or welfare services related to housing, social security schemes, education, etc., probably reflects the fact that these were readily available to our informants. When, on the other hand, these were not available, Jones et al. [10] found that the perception of ‘social vulnerability’ was a serious barrier to continuity of treatment.

## Lack of integration between specialist services

Our study shows that the pathways of the informants with problems of addiction were characterised by frequent transfers between specialist services, as a consequence of which difficulty was most in evidence at any given time; addiction, anxiety, eating disturbances, etc. The result was a fragmented treatment pathway in which, at worst, individual problems might escalate because they had not been dealt with. The challenge facing these services is paradoxical because while specialised support services are provided, the need to look at these problems in a holistic manner is widely recognised.

The lack of integrated provision of services and simultaneous treatment is a well-known problem within the system, particularly where users with simultaneous mental and addiction problems are concerned. This is in spite of the fact that it has long since been documented that problems of mental health and addiction ought to be treated simultaneously and within the same system [32,33] and that treatment should be both uninterrupted and long term [34]. The majority of the persons who are treated in the addiction sector need simultaneous treatment for mental illness [35].

## Lack of progress in care

All of our informants had an offer of treatment by the specialist health services during part of or the entire the data-gathering period; relatively recently for the latest service users, extremely variable in the case of those with both addiction and mental health issues and stable over time in the group who had no problems of addiction. The pathways of three out of the four in the last group were marked by development and improvement; in two cases also by active reductions in treatment. For these users, the treatment regimes had been, and still were, characterised by aims and direction, and the users reported that their level and quality were adapted to their situation and needs. The exception was Mona, who described a pathway with regular support conversations in the primary care services and acute hospital admissions when her condition worsened, which happened about once a year. At her own request for measures that could improve her condition, she was provided with out-patient specialist treatment.

Users who find themselves at the ‘wrong service level’ have been described as a widespread problem [22], first and foremost when the patient has been admitted to a day-care institution, but ought rather to have had an offer of treatment within primary care. One study has shown that in Norway, this is the situation for one-third of such patients, as evaluated by their treatment providers [36]. However, another study found that one out of every three adult users who were followed up exclusively within primary care, required specialist treatment, as primary care personnel evaluated the situation [26]. Being left at the wrong service level, whether primary or secondary, indicates that the pathway is stagnant - i.e. a ‘cementation’. The different administrative levels of primary and secondary care services probably lead to bureaucratic or attitudinal barriers or blockages being created and maintained. Generally speaking, the suitability of the range of services ought to be more important than whether users find themselves at the ‘right’ level in administrative terms, but when the organisation itself creates barriers, the focus is naturally on this aspect. The central aim must be to attempt to provide services and measures that improve the user's situation that lead to progress and development in the pathway of the patient or user. The ‘balanced approach’ of Thornicroft and Tansella [30,31] could provide a guiding line in this respect. Concrete proposals on a national level involve honing the specialist level into taking care of that which is rare, especially knowledge-intensive or challenging or simply to remove this level [37].

## Inadequate coordinating tools

Most of our informants had some kind of coordination measure in the form of case conferences, ‘Individual Plan’, a regular contact person or intermittent follow-up by a team. Studies show that teamwork and case management lead to continuity [38,39]. Hence, these tools must bear some of the responsibility for the lack of integration between the various specialist services.

Even if ‘Individual Plan’ is a statutory right, and various studies point to problems related to the crosswise implementation of services, administration, laws and professions [40–42]. Vold Hansen [40] explains this as an existing mismatch between the rational-instrumental logic that the ‘Individual Plan’ is arising out of and the sector's complexity. Difficulties in implementation are also linked to the lack of participation from involved services, first and foremost the general practitioners [43]. It is also claimed that more user involvement is needed for greater effectiveness [42]. User interaction requires that the user trust the clinician/helper, something that requires relationship building [44], and with a good relationship the ‘Individual Plan’ may have ‘elements of therapy in itself’ [44, p. 19].

## A more proactive continuity of care concept

Traditionally, an important aim of continuity of care within the mental health services has been to prevent dropout [6]. This is a relevant perspective vis-à-vis users who are reluctant to engage with follow-up procedures, but it is not very proactive. Sweeney et al. [8] claim that the continuity of care literature tends to overlook users' ‘desire for services that help them to move forward’. Our findings also indicated that the informants were not met by well-coordinated services that accepted their ambitions regarding their own care pathway. More user-oriented as well as more ambitious services could have provided a better basis for the discussion of possibilities and aims with users in order to prepare the groundwork for implementing the most appropriate measures. If the continuity of care concept is to be of widespread importance, the concept will have to include a more proactive perspective regarding how progress and development can be created in the users' care pathways within the system of mental health services. Our findings also suggest that such a perspective is of relevance to users with diagnoses of both severe and less-severe illnesses.

In a critical synthesis of literature on continuity of care in England in 2000–9, Heaton et al. [16] reveal indications of an emerging shift from the patient and carer ‘perspectivist’ paradigm towards a more dynamic ‘partnership’ paradigm where continuity is recognised to be co-constructed by patients, families and professionals, all of whom have an active part to play in its accomplishment.

Continuity of care in a mental health service user's pathway has both an organisational rationale; the orderly, uninterrupted movement of patients [1], and experiential considerations; health care events experienced as coherent, connected, and consistent with the user's healthcare needs and personal context [4]. In this study, we have explored and identified failures in the first of these two facets, i.e. the movement and progress of users within the system. This article will be followed up by a companion article that will summarise the results regarding experiential considerations among the informants.

## Conclusions

Continuity of care was hindered by the system when the user was either stuck in the queue for treatment, continually alternating between different specialist services or stuck in services without sufficient progress, while established coordinating measures did not prevent this from happening. The objectives of continuity of care should be more closely linked to aspirations for development and progress in mental health service users' care pathways. At the same time, one should display sensitivity vis-à-vis users who are less ambitious with regard to development and progress in their own care pathway. This approach coupled with good tools for coordination, where the user and the services involved together raise the bar for further work, is probably the most effective way to counteract the system barriers that exist. Well-known and widely used coordination tools such as ‘Individual Plan’ and case conferences should be upgraded to this end and utilised to the utmost.

## Figures and Tables

**Table 1.**
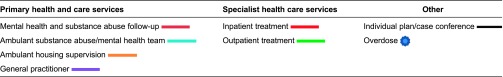
Categories of the various services mentioned in Figures 1–3
